# Thoracic Lymphatic Perfusion Patterns Assessed by Magnetic Resonance Imaging and Late Fontan Failure

**DOI:** 10.3390/diagnostics14232611

**Published:** 2024-11-21

**Authors:** Diego B. Ortega-Zhindón, Gabriela Meléndez-Ramírez, Sergio A. Patrón-Chi, Frida Rivera-Buendía, Juan Calderón-Colmenero, José A. García-Montes, Nonanzit Pérez-Hernández, José Manuel Rodríguez-Pérez, Jorge L. Cervantes-Salazar

**Affiliations:** 1 Department of Pediatric Cardiac Surgery and Congenital Heart Disease, Instituto Nacional de Cardiología Ignacio Chávez, Mexico City 14080, Mexico; diegob.ortegaz@gmail.com; 2 Facultad de Medicina, Programa de Maestría y Doctorado en Ciencias Médicas, Odontológicas y de la Salud, Universidad Nacional Autónoma de México, Mexico City 045510, Mexico; drsergiopatron@gmail.com; 3 Department of Magnetic Resonance Imaging, Instituto Nacional de Cardiología Ignacio Chávez, Mexico City 14080, Mexico; gabrielamelram@yahoo.com.mx; 4 Consorcio de Investigación en Salud (CISIDAT AC), Cuernavaca 62000, Mexico; frida.rivera06@gmail.com; 5 Department of Pediatric Cardiology, Instituto Nacional de Cardiología Ignacio Chávez, Mexico City 14080, Mexico; juanecalderon@yahoo.com.mx; 6 Department of Interventional Cardiology in Congenital Heart Disease, Instituto Nacional de Cardiología Ignacio Chávez, Mexico City 14080, Mexico; pepegamon@yahoo.com.mx; 7 Department of Molecular Biology, Instituto Nacional de Cardiología Ignacio Chávez, Mexico City 14080, Mexico; unicanona@yahoo.com.mx (N.P.-H.); josemanuel_rodriguezperez@yahoo.com.mx (J.M.R.-P.)

**Keywords:** Fontan procedure, cardiovascular magnetic resonance imaging, lymphatic vessel, congenital heart disease

## Abstract

Background: Fontan circulation maintains an elevated venous pressure; this promotes venous and lymphatic congestion and may lead to late circuit failure. Our objective was to determine the association between thoracic lymphatic perfusion patterns assessed by magnetic resonance imaging and late Fontan failure. Methodology: A retrospective study was performed. We included patients who underwent the Fontan procedure between January 2005 and December 2019 and who were evaluated with lymphatic mapping using magnetic resonance imaging. Lymphatic abnormalities were classified into four types. The prevalence of late failure was determined, and logistic regression analysis was performed to establish the association between the variables of interest and the outcome. Results: Fifty-four patients were included with a mean age at surgery of 8.8 years ± 3.5 years; 42.6% (n = 23) were men. The most frequent diagnosis was tricuspid atresia (50%, n = 27), and the Fontan procedures were mainly performed using an extracardiac conduit (96.3%, n = 52). The prevalence of late Fontan failure was 35.2%. The lymphatic perfusion patterns observed were Type 1 in 25.9% (n = 14), Type 2 in 46.3% (n = 25), Type 3 in 25.9% (n = 14), and Type 4 in 1.8% (n = 1), with no differences in relation to late failure. (*p* = 0.42). The age at surgery was found to be a factor associated with the late Fontan failure (OR: 1.23; 95% CI: 1.02–1.48; *p* = 0.02). Conclusions: One-third of patients with Fontan circulation may experience late failure, not significantly associated with lymphatic changes, but when the total cavopulmonary connection is completed at an older age.

## 1. Introduction

Congenital heart disease accounts for one third of all congenital malformations, making them the most common birth defects worldwide, affecting up to 1% of newborns [1,2,3,4]. Due to regional variability, the prevalence varies between 2.1 and 12.3 per 1000 live births [1,2,4,5], and its incidence ranges from 6 to 8 per 1000 live births [1,2,6]. A particular population affected by a complex cardiac anomaly known as univentricular heart has been identified, where the estimated prevalence is 1 in 10,000 live births [7,8]; the existence of only one functional ventricle [9] poses a challenge in diagnosis, treatment, and follow-up due to the heterogeneous defects that preclude a biventricular repair.

Fifty years ago, a surgical treatment was described to palliate this group of patients with univentricular heart [10,11], having several modifications [7,12,13,14,15] that sought to mimic a favorable hemodynamic circuit; since having a parallel circulation, the objective was to separate the systemic and pulmonary circulation, to avoid a volume overload in the single ventricle, as well as to derive the systemic venous return directly to the pulmonary arteries, allowing a passive flow to the lungs without a subpulmonary ventricle, thus establishing a serial circulation [7,16,17,18]. A mean survival of two to three decades after the surgery occurs for 70% to 85% of individuals who undergo this surgical procedure [3,4,19,20]. In this regard, it is estimated that there are between 47,000 and 70,000 living patients who had the Fontan procedure worldwide [7,21,22], and it is crucial to consider that 20% to 40% of individuals with a single ventricle who had this palliative surgery could experience a late failure of the “new circuit” in 20 to 25 years [22,23,24].

Chronic and permanent exposure to different hemodynamic changes characterized by an elevated central venous pressure and a low cardiac output [16,17,18] compromises the lymphatic system, which is part of the cardiovascular system. This impairment limits the capacity of the lymphatic system to regulate fluid homeostasis, which could lead to lymphatic insufficiency manifested by pleural effusion, ascites, protein-losing enteropathy (PLE), or plastic bronchitis (PB) [21,25,26,27,28,29]; there is the importance of identifying the hemodynamic deterioration in a timely manner. Nowadays, understanding of the role of the lymphatic system in the Fontan circulation, along with the development of lymphatic imaging techniques using magnetic resonance imaging (MRI) [30,31,32,33,34], has facilitated the evaluation of the anatomical changes that occur due to an altered lymphovenous pressure gradient secondary to central venous congestion [25,26,27,28,29]. This allows the establishment of potential diagnostic markers that anticipate late failure and avoid clinical stages of no return (Fontan takedown, heart transplant, and/or death).

Therefore, the aim of this study was to determine the association between thoracic lymphatic perfusion patterns assessed by magnetic resonance imaging and late Fontan failure.

## 2. Materials and Methods

A retrospective, observational, and analytical study was conducted with patients under 18 years of age who underwent the Fontan procedure at the Instituto Nacional de Cardiología Ignacio Chávez from 1 January 2005 to 31 December 2019. The participants had to be alive, without a Fontan takedown, and have had at least one follow-up MRI study. We excluded patients who underwent the Fontan procedure at another institution (n = 8), those who were not located or did not attend their follow-up appointments (n = 71), as well as patients with suboptimal MRI studies or no MRI performed (n = 17), dead patients (n = 19), and those who had Fontan takedown (n = 12). The variables were collected from the clinical records. The study was approved by the local institutional review board (CI-009-2023), and a waiver of informed consent was granted.

We gathered demographic data, main diagnoses, predominant ventricular morphology, preoperative and postoperative functional class according to the New York Heart Classification Association (NYHA) [35], surgical history, and cardiac catheterization history, as well as preoperative anatomic and hemodynamic characteristics assessed by echocardiography and cardiac catheterization. The surgical variables were the use and time length of cardiopulmonary bypass (CPB), the use and time length of aortic clamping, the type of cardioplegia, the conduit size, and the fenestration size. The postoperative and follow-up variables were the length of stay in the pediatric intensive care unit (PICU); the time length of mechanical ventilation; the time length and volume of thoracic and peritoneal drainages; the hemodynamic characteristics assessed by echocardiography, cardiac catheterization, and MRI; as well as early and late postoperative complications.

A late Fontan failure was considered when one or more of the following conditions appeared after discharge and during follow-up: peripheral edema, pleural effusion, ascites, PB, and PLE. Peripheral edema was defined as the presence of pitting (regardless of the degree of intensity) in the lower extremities in a symmetrical manner, which was assessed by a clinical cardiologist during the follow-up [36,37]. Pleural effusion was defined as the presence of pleural fluid assessed by MRI, while ascites was defined as the presence of abdominal fluid detected by abdominal ultrasound. PB was determined with the expectoration of bronchial-tree-shaped casts, associated or not with respiratory distress or airway obstruction [21]. PLE was considered when one or more of the following clinical data were present: edema, abdominal discomfort, diarrhea, or effusions (ascites, pleural, or pericardial effusions); associated with decreased serum albumin (<3.5 g/dL) or decreased total serum protein levels (<6 g/dL); and confirmed by intestinal protein loss (increased fecal α1-antitrypsin > 54 mg/dL or α1-antitrypsin clearance > 27 mL/24 h without diarrhea and >56 mL/24 h with diarrhea) [21,38,39,40]. The primary outcome was the late Fontan failure.

### 2.1. Evaluation Protocol of the Lymphatic System by Magnetic Resonance Imaging

The studies were performed on a 1.5 T scanner (Magnetom Avanto; Siemens Healthcare, Berlin, Germany) at the Department of Magnetic Resonance Imaging of the Instituto Nacional de Cardiología Ignacio Chávez. The previously standardized imaging protocol [31,32,33] gathered lymphatic mapping with a T2-weighted MRI using a three-dimensional turbo spin-echo sequence. The respiratory and cardiac navigation parameters were a coronal orientation matrix of 256 × 256, a field of view of 300 to 450, a repetition time of 2500 ms, an echo time of 650 ms, a flip angle of 140°, and a voxel size of 1.1 × 1.1 × 1.1 mm. The scanning time was between 4 and 8 min, depending on the patient size. Every imaging study included the neck, thorax, and abdomen. No contrast medium was used in any study. The sequences used allowed the creation of a three-dimensional array that was formatted in maximum intensity projection reconstructions for the coronal plane and customized from the source images. The image processing was performed in commercial software (Syngo InSpace Dynamic; Siemens Healthcare, Germany).

### 2.2. Assessment and Classification of the Thoracic Lymphatic Perfusion Patterns

Images of the coronal plane were analyzed independently by two cardiovascular imaging specialists: a cardiologist (GMR) and a pediatric cardiologist (SAPC); both were unaware of the clinical and surgical characteristics of the patients. The thoracic lymphatic perfusion patterns were classified separately; discrepancies between the 2 reviewers were discussed together and with a third evaluator specialized in congenital heart surgery (DBOZ) until a final decision was reached.

**Figure 1:**
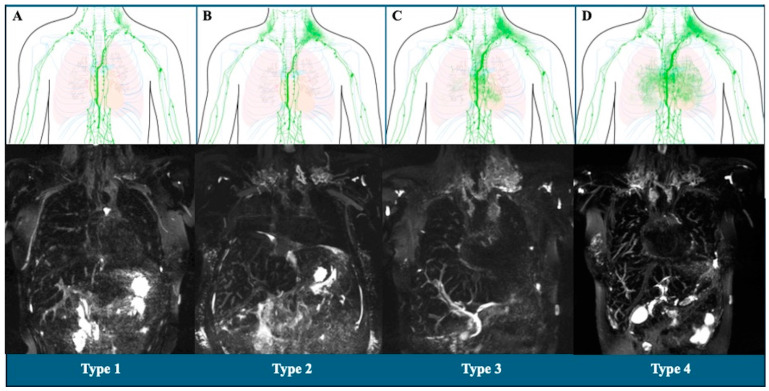
Schematic and magnetic resonance imaging representations of the thoracic lymphatic perfusion patterns. (**A**): Minimal changes in the supraclavicular region (Type 1); (**B**): bilateral changes in the supraclavicular region (Type 2); (**C**): changes in the supraclavicular region with extension to the mediastinum (Type 3); and (**D**): changes in the supraclavicular region with extension to the mediastinum and lung parenchyma regions (Type 4).

The thoracic lymphatic perfusion patterns were classified according to the reports by Biko et al., Ghosh et al., and Kelly et al. [31,32,34], considering 4 types of patterns depending on the involvement of the following regions: supraclavicular, mediastinum, and lung parenchyma (Figure 1). Type 1: when the changes at the supraclavicular level were minimal or without any alteration; type 2: when there were clear changes in the supraclavicular region, but without involving the mediastinum or lung parenchyma [31,32,34]; these two types were considered as low-grade lymphatic changes [34]. On the other hand, high-grade lymphatic changes were considered Types 3 and 4 [34]; Type 3 was when supraclavicular abnormalities were present, extending to the mediastinal region, but without reaching the lung parenchyma; the Type 4 pattern was when, in addition to the abnormalities in the supraclavicular and mediastinal regions, other abnormalities were observed in the lung parenchyma [31,32,34].

### 2.3. Statistical Analysis

We performed a descriptive analysis of the variables of interest. The categorical variables were described using frequencies and percentages. The quantitative variables were described using mean (standard deviation) or median (interquartile range), according to their distribution; this distribution was determined graphically, using histograms and QQ-plots; additionally, the asymmetry and kurtosis test (SK test) and the Shapiro–Wilk test were also performed. The interobserver variability was calculated using Cohen’s kappa coefficient (κ). A κ statistical coefficient between 0.61 and 0.80 suggested substantial agreement, while a value equal to or greater than 0.81 indicated excellent agreement.

The comparative analysis of the distribution of variables between the patients who had a late failure and those who did not included the X^2^ test or Fisher’s exact test performed for categorical variables according to the expected number of observations in each cell of the contingency tables, while the quantitative variables were compared using the *t*-Student test or the Mann–Whitney U test, according to their distribution. Univariate and multivariate logistic regression analyses were performed to determine the association between the variables of interest and the outcome. The magnitude of the effect was expressed by odds ratio and 95% confidence intervals. All tests were two-tailed. The Stata software version 17 (StataCorp LLC., College Station, TX, USA) was used for the analysis.

## 3. Results

### 3.1. Clinical–Demographic Characteristics

Between January 2005 and December 2019, 5100 surgeries were performed at our center, of which 3.4% (173) corresponded to the Fontan procedure. Fifty-four patients who met the inclusion criteria for this study were identified; 42.6% (n = 23) were men and 57.4% (n = 31) were women. At the time of surgery, the mean age was 8.8 ± 3.5 years, with a median weight of 24 kg (IQR 17.8–37) and a median body surface area of 0.9 m^2^ (IQR 0.7–1.7); no statistical differences between patients with and without late Fontan failure were observed (Table 1). During the preoperative stage, 77.8% (n = 42) of the patients were in NYHA functional class II, while 1.8% (n = 1) were in NYHA functional class IV (Table 1).

The main diagnoses included tricuspid atresia (50%, n = 27) and pulmonary atresia with intact interventricular septum (22.2%, n = 12), while the double-outlet right ventricle, transposition of the great arteries, and other heart conditions were less frequent (Table 2). Forty-eight patients (88.9%) had a history of a previous surgery, and 17 (31.5%) had at least one second intervention, with the main procedures being systemic-to-pulmonary shunt in 28 and bidirectional cavopulmonary connection in 29 cases. (Table 1 and Table 2). As part of the surgical protocol, all patients had cardiac catheterizations before surgery; however, in 9 (16.7%) patients, a second cardiac catheterization before surgery was documented.

Among the anatomical and hemodynamic characteristics observed before surgery, the predominant ventricular morphology was left (75.9%, n = 41), less frequently right (16.7%, n = 9), and undetermined (7.4%, n = 4); despite these findings, there were no important differences (*p* = 0.17) (Table 1). Other characteristics were also considered, and we observed that the mean arterial oxygen saturation was 75.4 ± 6.8%, with a median systemic ventricular ejection fraction of 61.3% (IQR 60–65), with no difference between patients with and without failure (Table 1). When evaluating the fundamental hemodynamic conditions, we observed that the mean systemic ventricular end-diastolic pressure (SVEDP) was 8.4 ± 1.8 mmHg, while the mean pulmonary artery pressure (mPAP) was 11.7 ± 2.3 mmHg. When comparing patients with and without failure, both SVEDP (*p* = 0.96) and mPAP (*p* = 0.79) showed no significant differences (Table 1).

### 3.2. Surgical Characteristics

The Fontan procedure was performed in 51.9% (n = 28) of the cases with CPB; of these, 39.2% (n = 11) were performed with aortic clamping (Table 3). In the latter cases, an antegrade crystalloid cardioplegia (Custodiol^®^) was used as myocardial protection in 9 (32.1%) cases, while del Nido cardioplegia was used in the other two (7.1%) patients, with no differences between those who had CPB and those who did not (*p* = 0.07). In this sense, it was observed that the median CPB time length was 129 min (IQR 96–155.5), with no significant differences between patients with and without failure (*p* = 0.38). Similarly, the median aortic clamping time length was 83 min (IQR 15–110), also without differences regarding having or not having late failure (*p* = 0.85) (Table 3).

In 52 (96.3%) patients, the Fontan procedure was performed with an extracardiac conduit; the mean conduit size was 18.3 ± 1.7 mm, with very similar values for both groups (*p* = 0.15). In all cases, a fenestra was left, with a mean size of 7.1 ± 1.7 mm, with a slight decrease in size in patients who had a failure, but without representing a significant difference (7.2 ± 1.5 without a failure vs. 6.8 ± 1.8 with failure) (Table 3).

### 3.3. Early Results

The median length of stay in PICU was 4.9 days (IQR 2.9–7.8), with a median time length on mechanical ventilation of 29.8 h (IQR 15–72); these values were similar between patients with and without failure (Table 3). Likewise, the postoperative hemodynamic behavior was very similar between patients with and without a failure, showing a mean arterial oxygen saturation of 89.1 ± 5.7 % (*p* = 0.11), a median systemic ventricular ejection fraction of 60% (IQR 56–61) (*p* = 0.08), mean SVEDP measured through the mean left atrial pressure of 9.9 ± 1.9 mmHg (*p* = 0.53), and a mean mPAP of 14.4 ± 3.6 mmHg (*p* = 0.48) (Table 3).

Regarding the time length of chest tube drains, the median was 14.3 days (IQR 9.9–19.9); despite no significant differences observed between both groups (*p* = 0.92), patients with late failure showed a slight increase in time length (13.9 days (IQR 9.8–18.9) without failure vs. 16.9 days (IQR 8.9–20.8) with failure); however, when comparing the drained volume, there was an inversion between the groups (275 mL/m^2^/day (IQR 214–387) without failure vs. 256 mL/m^2^/day (IQR 231–376) with failure), but without statistical significance (*p* = 0.59) (Table 3). When assessing ascites through the use of a peritoneal dialysis catheter, the median time length of use was 10.9 days (IQR 7.9–15.9), with no significant differences between patients with and without failure (*p* = 0.83); when comparing the volume obtained between these patients, a lower volume was produced in patients with failure ((280 mL/m^2^/day (IQR 164–413) without failure vs. 266.5 mL/m^2^/day (IQR 194–392) with failure), but with no statistically significant differences (*p* = 0.96) (Table 3). The median hospital stay was 26.5 days (IQR 19–36).

### 3.4. Follow-Up and Late Fontan Failure

The follow-up time median was 8.6 years (IQR 6.1–11.9). When the MRI was performed, the patients’ mean age was 15.9 ± 2.9 years, with a significant difference (*p* = 0.006) between patients with and without late failure (16.8 ± 4.3 years without failure vs. 20.8 ± 5.9 years with failure), as well as a significant difference in body surface area (*p* = 0.03) (Table 3). Most cases (98.1%; n = 53) were in NYHA functional class II (Table 3).

The following conditions were observed during the follow-up: peripheral edema in 31.5% (n = 17), PB in 1.8% (n = 1), and PLE in 1.8% (n = 1). The prevalence of late Fontan failure was 35.2% (n = 19). Of these 19 patients, 15.8% (n = 3) presented moderate atrioventricular valve regurgitation, 5.2% (n = 1) presented severe atrioventricular valve regurgitation, and 79% (n = 15) had mild or no atrioventricular valve regurgitation; therefore, this condition was not significant (*p* = 0.39). When assessing the characteristics of the systemic ventricle by means of MRI, a median ventricular ejection fraction of 52% (IQR 47–56) was observed, with a median indexed end-systolic volume of 35.9 mL/m^2^ (IQR 26.9–42.1) and a mean indexed end-diastolic volume of 74 ± 28.9 mL/m^2^ with no significant differences between both groups (Table 3).

### 3.5. Thoracic Lymphatic Perfusion Pattern and Late Fontan Failure

When evaluating the lymphatic perfusion pattern, 25.9% (n = 14) of Type 1, 46.3% (n = 25) of Type 2, 25.9% (n = 14) of Type 3, and 1.8% (n = 1) of Type 4 were found, with no significant differences (*p* = 0.42) (Table 3). The type 2 was prevalent in both groups, with failure (36.8%, n = 7) and those without failure (51.4%, n = 18). Patients with late failure also presented high-grade lymphatic changes (Types 3 and 4) in up to 31.6% of cases (n = 6) (Table 3). In this context, when evaluated according to lymphatic perfusion patterns and follow-up period, patients with a high grade presented a significant difference (*p* = 0.04), with longer follow-up being observed in those who experienced failure (Table 3).

The univariate analysis was performed to identify factors associated with late failure, such as age and weight at the time of surgery, ventricular dominance, Nakata index and McGoon ratio, use of CPB and aortic cross-clamping, systemic ventricular ejection fraction, and lymphatic perfusion pattern, without finding significant associations (Table 4). Subsequently, a multivariate analysis was performed using logistic regression models, in which age at surgery (OR: 1.23; 95% CI: 1.02–1.48; *p* = 0.02) was found to be an independent factor associated with late Fontan failure, while other variables of interest, including the lymphatic perfusion pattern, were discarded (Table 5).

## 4. Discussion

Patients with single ventricles have benefited from the Fontan procedure, giving them better long-term survival. However, changes in this complex circulation, which is chronically affecting circulatory homeostasis, take place sometimes; therefore, the medical team in charge of this population should regularly assess the physiological mechanisms and adaptations that occur in this new circuit, both in early and late phases. In our center, we have previously reported some results [41,42,43] with the aim of establishing and improving their care. One key component is to determine an adequate preoperative hemodynamic profile [43], which has been described and implemented for the past 20 years. In this context, the present study not only sought to determine the prevalence of patients with late failure but also to identify, through advanced MRI techniques, alterations in the lymphatic system that could be associated with a late Fontan failure.

Characterizing a late Fontan failure is highly complicated due to its multisystem component that accompanies the morbidity of these patients [21,44]. Therefore, we consider that the lymphatic system impairment not only occurs acutely, as other groups have pointed out [30,31,32,33,34], but it can manifest in advanced stages as well, producing a variety of signs and symptoms from mild conditions (such as edema) to more complex entities (such as PB and PLE) [26,27,28,29,38,39,40].

In the present study, the prevalence of late Fontan failure was 35.2%, and the main abnormalities of the lymphatic system observed were of low grade (Table 3). In turn, among the patients with high-grade lymphatic abnormalities, two cases showed severe complications such as PB and PLE; this contrasts with other series that have follow-ups longer than 6 months [33], where no severe complications were observed, but there were abnormalities in the lymphatic perfusion patterns as well as lymphatic congestion at different levels, both in the thoracic and abdominal regions [33]. On the other hand, our results are consistent with those reported by Ghosh et al. and Kelly et al. [32,34], who observed high-grade lymphatic perfusion patterns in early stages and follow-ups, along with severe conditions such as PB and PLE, suggesting a larger disruption of the lymphatic system due to the increased pressure resulting from a lymphovenous congestion [25,26,27,28,29].

It is necessary to point out some differences among the studies aforementioned. The mean age reported for the Fontan procedure was between 3 and 4 years [31,32,33,34]; in contrast, our results showed a mean age of 8 years (Table 1). Although no significant differences were found between patients with and without late failure, both in our series and in those previously reported [31,32,33,34], patients with a late Fontan failure were older. Thus, in the multivariate analysis of our series (Table 5), older age was the only characteristic associated with late Fontan failure (OR: 1.23; 95% CI: 1.02–1.48; *p* = 0.02).

Furthermore, an important difference between the findings of the North American and European groups [32,33,34] and our study lies in the higher prevalence of patients with right-predominant ventricular morphology they observed, while in our center, the left-predominant ventricular morphology (Table 1). This is because the main pathology we reported is the tricuspid atresia (Table 2), a condition that we have observed since our first reports [41,43], while the North American and European groups mainly analyzed the hypoplastic left ventricle syndrome. Our results did not show a significant difference (*p* = 0.17) in relation to ventricular dominance, which is similar to what Dittrich et al. [33] found (*p* = 0.71); however, Ghosh et al. [32] observed a difference based on this condition (*p* = 0.047).

In the postoperative period, we evaluated both the volume of pleural effusion and the time length of chest tube drainage. Regarding pleural effusion, there were no differences (*p* = 0.96) in volume between patients with and without late (Table 3), but a significant reduction in pleural effusion compared to previous reports [43], where pleural effusion was significantly different between patients who survived and those who died.

Regarding the time length of chest tube drainage, our findings are similar to those reported by Dittrich et al. [33], with no significant differences between patients with and without failure; however, a longer duration in days of chest drainage was observed in patients with failure (Table 3). In turn, Kelly et al. [34] found differences (*p* < 0.001) in patients with failure, associating the prolonged use of chest tube drainage with the progression to a high-grade lymphatic perfusion pattern, for these structural anomalies are dynamic; in our case, we believe a new control is necessary to demonstrate this association during follow-up.

Taking into consideration that 20% to 40% of patients who undergo the Fontan procedure may experience some degree of Fontan failure in the first twenty years [22,23,24], and having a mean follow-up of 8.6 years in our center, we analyzed the systemic ventricular ejection fraction as a characteristic susceptible to alteration in these patients, which could be related to hemodynamic deterioration. This was based on the fact that a chronic and insufficient preload of the systemic ventricle may contribute to the development of ventricular dysfunction [24]; nevertheless, in the univariate and multivariate analyses, no such association was found (Table 4 and Table 5).

At the same time, other characteristics of the systemic ventricle were assessed, comparing patients with and without late Fontan failure; we did not observe differences in systolic or diastolic ventricular volumes, nor in the degree of atrioventricular valve regurgitation (Table 3), unlike what was reported by Sallmon et al. [24], which in a mean follow-up of 20 years suggested that late Fontan failure is caused by ventricular dysfunction before the increased in pulmonary vascular resistance, which deteriorates over time in response to the hemodynamic component involving preload, filling pressures, and pulmonary blood flow among others; therefore, an analysis should be considered in our series with a more extensive follow-up.

As previously mentioned, our group has maintained a “strict” approach to patient selection over the past two decades, considering their hemodynamic profile [41,43], with the aims of reducing the incidence of early complications and improving survival; therefore, we sought to analyze variables that could be factors associated with severe complications such as PB and PLE, as well as other less serious complications such as pleural effusions and ascites, that can significantly affect the quality of life of patients. Two basic hemodynamic parameters were analyzed: SVEDP and mPAP. These parameters showed no differences between patients with and without failure in both the preoperative and immediate postoperative periods (Table 1 and Table 3). This contrasts with the findings by Kelly et al. [34] and by Sallmon et al. [24], who reported significant changes in these variables in patients with failure. Despite these results, we agree with Sallmon et al. [24] that not only these conditions will determine adequate ventricular functioning, but it is necessary to consider a set of hemodynamic factors, together with pharmacological support. In our case, the test of time remains to establish a similarity or difference with this study.

Ultimately, in our patients, we did not find any association between late Fontan failure and thoracic lymphatic perfusion patterns (Table 4 and Table 5), contrary to what was described by Ghosh et al. [32], where patients with high-grade perfusion patterns in the early postoperative stage were more likely to develop complications than those with low-grade patterns (OR: 6.28; 95% CI: 2.13–18.5; *p* = 0.001). However, when analyzing patients with high-grade lymphatic changes, those with longer follow-up showed significant differences in the presence of late failure compared to those who did not (Table 3), so we agree with Dittrich et al. [33] and Kelly et al. [34] that long-term and routine follow-ups are essential at all stages of the Fontan procedure, given the dynamic physiology observed in these patients, in order to determine in the future the possible association between lymphatic perfusion patterns and the late Fontan failure.

We recognize the limitations of our study. The characteristic retrospective, single-center, non-randomized study limits the generalizability of our findings. In addition, although we collected a complete set of variables to analyze, there may be other variables that were not considered for the outcome; in the future, we would like to be able to contrast the late Fontan failure with the lymphatic changes and their causal determinants. Likewise, the limited number of patients; given the recent implementation of this protocol using MRI for evaluation, we could not include a larger number of cases, in addition to the fact that being a third-level center, some patients are followed up in their primary contact centers due to demographic, social, and economic constraints that prevent them from transferring to our center. In addition, we believe that further analysis with long-term follow-up will be essential since the results obtained suggest that the time factor and the duration of follow-up will be determinants of future results. Despite these limitations, we consider that this first report offers valuable information for clinical and surgical groups on what happens with patients who undergo the Fontan procedure, mainly in our country and region.

## 5. Conclusions

Our study revealed that approximately one-third of patients who underwent the Fontan procedure during the follow-up phase may experience late failure. This condition is associated with advanced age at the time the total cavopulmonary connection was completed, although no significant relationship with lymphatic changes was established. This forces us to create evaluation protocols in the preoperative, postoperative, and follow-up stages to promptly identify a late Fontan failure and thus establish adequate treatment before reaching the final stages (heart transplant or Fontan takedown), improving the survival and quality of life of these patients.

## Figures and Tables

**Table 1:** Overall characteristics of patient with and without late Fontan failure.

Characteristics, n (%)	Total 54 (100)	With Late Failure 19 (35.2)	Without Late Failure 35 (64.8)	*p*
Sex, n (%)				
Male	23 (42.6)	9 (47.4)	14 (40)	0.61
Female	31 (57.4)	10 (52.6)	21 (60)	
Age at surgery (years), mean ± SD	8.8 ± 3.5	10.1 ± 3.4	8.2 ± 3.4	0.06
Weight at surgery (kg), median (IQR)	24 (17.8–37)	30 (19.2–44.5)	21 (16.5–36.5)	0.09
BSA at surgery (m^2^), median (IQR)	0.9 (0.7–1.2)	1.05 (0.7–1.4)	0.85 (0.71–1.2)	0.26
Preoperative NYHA class, n (%)				
I	2 (3.7)	1 (5.2)	1 (2.8)	0.76
II	42 (77.8)	14 (73.7)	28 (80)	
III	9 (16.7)	4 (21.1)	5 (14.4)	
IV	1 (1.8)	0 (0)	1 (2.8)	
Previous surgeries, n (%)				
1	48 (88.9)	16 (84.2)	32 (91.4)	0.42
2	17 (31.5)	6 (31.6)	11 (31.4)	0.99
3	1 (1.8)	1 (5.2)	0 (0)	-
Pre-Fontan surgical procedures, n (%)				
Systemic-to-pulmonary shunt	28 (51.8)	10 (52.6)	18 (51.4)	0.93
Bidirectional cavopulmonary connection	29 (53.7)	9 (47.4)	20 (57.1)	0.49
Predominant ventricular morphology, n (%)				
Left ventricle	41 (75.9)	14 (73.7)	27 (77.1)	0.17
Right ventricle	9 (16.7)	2 (10.5)	7 (20)	
Undetermined	4 (7.4)	3 (15.8)	1 (2.8)	
Pre-operative anatomical/hemodynamic conditions				
Arterial oxygen saturation (%), mean ± SD	75.4 ± 6.8	73.6 ± 6.9	76.4 ± 6.6	0.15
* Systemic ventricular ejection fraction (%), median (IQR)	61.3 (60–65)	62 (61–64.2)	61 (57.6–67)	0.41
Systemic ventricular end-diastolic pressure (mmHg), mean ± SD	8.4 ± 1.8	8.4 ± 2.2	8.4 ± 1.5	0.96
Mean pulmonary artery pressure (mmHg), mean ± SD	11.7 ± 2.3	11.6 ± 2.1	11.8 ± 2.4	0.79
Nakata index (mm^2^/m^2^), median (IQR)	242.5 (200–304)	239 (203–328)	246 (183–300)	0.47
McGoon ratio, mean ± SD	2.1 ± 0.3	2.03 ± 0.35	2.1 ± 0.3	0.65

BSA: body surface area, IQR: interquartile range, NYHA: New York Heart Association, and SD: standard deviation. * Assessed through a transthoracic echocardiogram.

**Table 2:** Diagnoses and pre-Fontan palliative procedures.

Characteristics, n (%)	Total 54 (100)
Diagnosis n (%)	
Tricuspid atresia	27 (50)
Pulmonary atresia with intact interventricular septum	12 (22.2)
DORV	6 (11.1)
TGA	3 (5.6)
Unbalanced AV channel	2 (3.7)
Ebstein’s anomaly	1 (1.8)
Others	3 (5.6)
Fontan palliative procedures n (%)	
Systemic-to-pulmonary shunt	28 (51.8)
Bidirectional cavopulmonary connection	29 (53.7)
Pulmonary artery banding	6 (11.1)
Damus-Kaye-Stansel procedure	1 (1.8)

AV: atrioventricular, DORV: double-outlet right ventricle, and TGA: transposition of the great arteries.

**Table 3:** Operative and postoperative characteristics of patients with and without late Fontan failure.

Characteristics, n (%)	Total 54 (100)	With Late Failure 19 (35.2)	No Late Failure 35 (64.8)	*p*
Surgical characteristics				
Surgical approach, n (%)				
With CPB	28 (51.9)	13 (68.4)	15 (42.9)	0.07
Without CPB	26 (48.1)	6 (31.6)	20 (57.1)	
CPB and aortic clamping				
Time length of CPB (min), median (IQR)	129 (96–155.5)	131 (106–199)	129 (87–153)	0.38
Time length of aortic clamping (min), median (IQR)	83 (15–110)	74.5 (31–98)	90 (14–112)	0.85
Cardioplegia type, n (%)				
Custodiol	9 (32.1)	5 (26.3)	4 (11.4)	0.77
del Nido	2 (7.1)	1 (5.2)	1 (2.8)	
Fontan type, n (%)				
Extracardiac TCPC	52 (96.3)	18 (94.8)	34 (97.2)	0.65
Intracardiac TCPC	2 (3.7)	1 (5.2)	1 (2.8)	
Conduit size (mm), mean ± SD	18.3 ± 1.7	18.6 ± 1.3	18.1 ± 1.02	0.15
Fenestra size (mm), mean ± SD	7.1 ± 1.7	6.8 ± 1.8	7.2 ± 1.5	0.47
Stay in PICU (days), median (IQR)	4.9 (2.9–7.8)	4.8 (2.8–7.8)	4.9 (2.9–7.9)	0.61
Mechanical ventilation time length (h), median (IQR)	29.8 (14–98)	27.5 (14–75)	34 (11.6–121.5)	0.62
Maximum lactate (mmol/L), median (IQR)	3.2 (2.2–4.5)	3.3 (2.2–4.9)	3.2 (2.1–4.1)	0.70
Post-operative characteristics				
Post-operative hemodynamic conditions				
Arterial oxygen saturation (%), mean ± SD	89.1 ± 5.7	87.4 ± 5.1	90 ± 5.8	0.11
* Systemic ventricular ejection fraction (%), median (IQR)	60 (56–61)	60 (55–62)	60 (55–61)	0.08
Mean left atrial pressure (mmHg), mean ± SD	9.9 ± 1.9	10.1 ± 2.1	9.8 ± 1.9	0.53
Mean pulmonary artery pressure (mmHg), mean ± SD	14.4 ± 3.6	14.8 ± 2.9	14.1 ± 3.9	0.48
Chest tube drains				
Drainage time (days), median (IQR)	14.3 (9.9–19.9)	16.9 (8.9–20.8)	13.9 (9.8–18.9)	0.92
Volume (mL/day/m^2^), median (IQR)	274 (227–376)	256 (231–376)	275 (214–387)	0.59
Peritoneal drainage				
Drainage time length (days), median (IQR)	10.9 (7.9–15.9)	10.8 (9.8–14.1)	10.6 (7.9–18.9)	0.83
Volume (mL/day/m^2^), median (IQR)	275 (175–411)	266.5 (194–392)	280 (164–413)	0.96
Follow-up characteristics				
Age (years) at MRI, mean ± SD	15.9 ± 2.9	20.8 ± 5.9	16.8 ± 4.3	0.006
Weight (kg) at MRI, mean ± SD	53.2 ± 14.3	58.3 ± 14.1	50.5 ± 14.1	0.05
BSA (m^2^) at MRI, mean ± SD	1.4 ± 0.2	1.5 ± 0.2	1.4 ± 0.2	0.03
NYHA class at follow-up, n (%)				
I	1 (1.8)	0 (0)	1 (2.8)	0.45
II	53 (98.1)	19 (100)	34 (97.2)	
Functional conditions assessed thorough MRI				
Atrioventricular valve regurgitation, n (%)				
None	31 (57.4)	10 (52.6)	21 (60)	0.39
Mild	16 (29.6)	5 (26.4)	11 (31.6)	
Moderate	4 (7.4)	3 (15.8)	1 (2.8)	
Severe	3 (5.6)	1 (5.2)	2 (5.6)	
** Systemic ventricular ejection fraction (%), median (IQR)	52 (47–56)	52 (46–56)	52 (47–56)	0.85
ESV indexed—systemic ventricle (mL/m^2^), median (IQR)	35.9 (26.9–42.1)	35.8 (31.4–43.2)	36 (25–41.9)	0.53
EDV indexed—systemic ventricle (mL/m^2^), mean ± SD	74 ± 28.9	81.5 ± 65.7	71.2 ± 26.4	0.89
Thoracic lymphatic perfusion pattern, n (%)				
Type 1	14 (25.9)	6 (31.6)	8 (22.8)	0.42
Type 2	25 (46.3)	7 (36.8)	18 (51.4)	
Type 3	14 (25.9)	5 (26.4)	9 (25.8)	
Type 4	1 (1.8)	1 (5.2)	0 (0)	
Follow-up after Fontan (years), median (IQR)				
Type 1	9.1 (5.9, 12.3)	11.8 (5.9, 13.3)	8.4 (5.5, 10.9)	0.49
Type 2	8.2 (6.2, 11.7)	10.6 (6.2, 17.3)	8.2 (6.1, 11.6)	0.42
Type 3 + 4	9 (5.8, 11.7)	11.8 (6.9, 12.3)	6.7 (5.2, 9.2)	0.04

BSA: body surface area, CPB: cardiopulmonary bypass, EDV: end-diastolic volume, ESV: end-systolic volume, IQR: interquartile range, MRI: magnetic resonance imaging, NYHA: New York Heart Association, PICU: pediatric intensive care unit, SD: standard deviation, and TCPC: total cavopulmonary connection. * Assessed through transthoracic echocardiogram, ** Assessed through magnetic resonance imaging.

**Table 4:** Univariate analysis of factors associated with late Fontan failure.

Variable	OR	IC 95%		*p*
		Low	High	
Age at surgery (years)	1.17	0.98	1.38	0.06
Weight at surgery (Kg)	1.04	0.99	1.08	0.11
Predominant ventricular morphology				
Left ventricle	Ref.	-	-	
Right ventricle	0.55	0.10	3.01	0.49
Undetermined	5.78	0.54	60.87	0.14
Nakata index (mm^2^/m^2^)	1.01	0.99	1.01	0.75
McGoon ratio	0.67	0.12	3.71	0.65
Use of CPB	0.34	0.11	1.12	0.07
Use of aortic clamping	1.71	0.37	7.91	0.49
** Systemic ventricular ejection fraction at follow-up (%)	1.09	0.95	1.25	0.19
Thoracic lymphatic perfusion pattern				
Type 1	Ref.	-	-	
Type 2	0.51	0.13	2.04	0.34
Types 3 + 4	0.88	0.20	3.91	0.87

CPB: cardiopulmonary bypass. ** Assessed through magnetic resonance imaging.

**Table 5:** Multivariate analysis of factors associated with late Fontan failure.

Variable	OR	IC 95%		*p*
		Low	High	
Age at surgery (years)	1.23	1.02	1.48	0.02
** Systemic ventricular ejection fraction at follow-up (%)	1.15	0.99	1.35	0.06
Thoracic lymphatic perfusion pattern				
Type 1	Ref.	-	-	
Type 2	0.52	0.12	2.31	0.39
Types 3 + 4	1.22	0.23	6.38	0.81

** Assessed through magnetic resonance imaging.

## Data Availability

Data supporting the results are available from the corresponding authors upon reasonable request.
